# Generalization of Motor Learning Depends on the History of Prior Action

**DOI:** 10.1371/journal.pbio.0040316

**Published:** 2006-09-12

**Authors:** John W Krakauer, Pietro Mazzoni, Ali Ghazizadeh, Roshni Ravindran, Reza Shadmehr

**Affiliations:** 1 Department of Neurology, Columbia University College of Physicians and Surgeons, New York, New York, United States of America; 2 Department of Biomedical Engineering, Johns Hopkins University, Baltimore, Maryland, United States of America; University of Minnesota, United States of America

## Abstract

Generalization of motor learning refers to our ability to apply what has been learned in one context to other contexts. When generalization is beneficial, it is termed transfer, and when it is detrimental, it is termed interference. Insight into the mechanism of generalization may be acquired from understanding why training transfers in some contexts but not others. However, identifying relevant contextual cues has proven surprisingly difficult, perhaps because the search has mainly been for cues that are explicit. We hypothesized instead that a relevant contextual cue is an implicit memory of action with a particular body part. To test this hypothesis we considered a task in which participants learned to control motion of a cursor under visuomotor rotation in two contexts: by moving their hand through motion of their shoulder and elbow, or through motion of their wrist. Use of these contextual cues led to three observations: First, in naive participants, learning in the wrist context was much faster than in the arm context. Second, generalization was asymmetric so that arm training benefited subsequent wrist training, but not vice versa. Third, in people who had prior wrist training, generalization from the arm to the wrist was blocked. That is, prior wrist training appeared to prevent both the interference and transfer that subsequent arm training should have caused. To explain the data, we posited that the learner collected statistics of contextual history: all upper arm movements also move the hand, but occasionally we move our hands without moving the upper arm. In a Bayesian framework, history of limb segment use strongly affects parameter uncertainty, which is a measure of the covariance of the contextual cues. This simple Bayesian prior dictated a generalization pattern that largely reproduced all three findings. For motor learning, generalization depends on context, which is determined by the statistics of how we have previously used the various parts of our limbs.

## Abstract 2

This human learning study indicates that a motor system learns not just from prediction error, but also from the history of implicitly remembered contexts in which training occurred.

## Introduction

Everyday experience suggests that we are able to learn multiple motor skills. In some situations, one skill can aid learning of another, but in other situations, we wish to recall one skill specifically without interference from other stored motor memories. For example, tennis players probably pick up table tennis faster than people who have never played racquet sports before. Indeed, it has been argued that the distinguishing feature of biological learning is generalization because our survival may depend on our ability to correctly extrapolate to contexts that are different from our limited experience [1]. Yet, generalization is a double-edged sword: if a small contextual change is associated with a large alteration of the learning problem, then generalization from prior learning will interfere with the new task, impair performance, and possibly catastrophically affect what was learned earlier. For example, when we drive in reverse, we have to do so slowly to avoid unwanted generalization from driving forward. In contrast, a stunt driver can learn and access models for forward and reverse driving independently.

In the past decade, numerous laboratories have been involved in quantifying patterns of generalization in motor learning, particularly in tasks that involve reaching. Two types of generalization have been addressed. First, the transfer component of generalization has been investigated by training in one context and then testing in another context, finding that transfer depends on the degree of contextual similarity between the training and test episodes [2–4]. For these tasks, context is often related to the state of the limb, such as the configuration or velocity of the arm [5]. Intriguingly, some generalization patterns are asymmetric. For example, learning to reach with prism goggles generalizes from arm motion to the wrist, but not vice versa [6,7]. Second, the interference component of generalization has been investigated by trying to train participants to acquire and recall opposite motor mappings. However, most experiments that have trained participants sequentially on two mappings, A and B, varying either the time between A and B and/or the number of alternations between A and B, have found flat gradients of persistent interference: mapping B appears to catastrophically interfere with mapping A, even with extended time intervals between them [8–11]. We have previously hypothesized that in these experiments the interference results from unwanted generalization because there is no change in context associated with the change from mapping A to mapping B [11].

In this study, we tested a series of hypotheses about the role of context in generalization of motor learning. We used an experimental paradigm that built on our previous finding that kinematics and dynamics are learned independently [12]. Specifically, a visuomotor rotation is learned separately from novel inertial dynamics. Our first hypothesis was therefore that the same rotation should transfer across different effectors, even though they have very different dynamics. The second hypothesis was that although rotation learning may be effector-independent, a change in effector would nevertheless serve as a powerful contextual cue to allow learning and recall of opposite rotations. The third hypothesis was that the degree to which learning generalizes between two contexts is not fixed but rather depends on the history of previous training in those two contexts.

In current theoretical approaches to motor learning, adaptation is viewed as a process in which prediction errors result in proportional changes in parameter estimates [13–16]. The mechanism of error-dependent change is the Rescorla-Wagner rule [17], also known as the “delta rule” or LMS (least means squared) rule, in which the generalization depends only on the contextual cues that are present. This computational framework assumes that generalization remains history invariant. Statistical models of learning provide an alternative way of thinking [18]. They emphasize both the prediction error and the uncertainty associated with parameter estimates. Critically, parameter uncertainty depends on the history of contexts, which in turn dictates generalization. For example, consider a classical conditioning task in which an animal learns to associate two different cues with a reward [19]. Suppose that a training set includes mostly instances in which both cues are present (say, a light and a tone). The animal learns that each cue predicts some fraction of the reward. However, it also accumulates information about the history of the trials and stores it in the uncertainty of the “weights” for each cue. As a result, when the reward is presented with only one cue, the statistical model predicts that while error should increase the weight associated with the present cue, it also should decrease the weight of the absent cue. That is, the animal generalizes the error to the unavailable contextual cue because in the past, the two cues appeared together [20]. Clearly, an animal that never observed the two cues together would have no reason to generalize prediction errors associated with one cue to the other.

Here we extend this statistical approach to the problem of motor learning, as a first step in understanding the origin of motor generalization. We first demonstrate that adaptation to a visuomotor rotation transfers from the arm to the wrist but not from the wrist to the arm. We then show that switching the limb segment used to move the hand can serve as a powerful contextual cue that allows learning of opposite visuomotor rotations in close temporal proximity. In effect, learning in a particular limb segment context can inhibit subsequent inter-segment generalization, resulting in the ability to maintain a different map for each context. We show that these results are supported by a single Bayesian model of motor learning in which generalization depends on the history of prior motor behavior.

## Results

Participants moved a cursor, which represented position of the tip of the index finger, to point to targets in two contexts. In the first, change in fingertip position was due to planar two-joint arm movements (wrist and fingers immobilized). In the second, change in fingertip position was due to movements of the wrist (shoulder and elbow immobilized). Our experimental goal was to show that the implicit memory of the effector used to learn a visuomotor rotation could serve as a contextual cue for recall.

### Experiment 1. Savings and Interference Occurred for Rotation Learning with the Wrist

“Savings” refers to the observation that performance during re-learning is better than initial learning. To establish that rotation learning at the wrist showed savings and interference in the same manner as previously reported for planar arm movements [12,21], we compared learning in three groups of participants. One group (group 1; Table 1) learned a 30° rotation at the wrist (R_wrist_) on day 1. The second group (group 2; Table 1) learned R_wrist_ on day 1 and then re-learned R_wrist_ 24 h later (day 2). This group showed savings, as re-learning of R_wrist_ on day 2 had considerably less error (Figure 1A). The third group (group 3; Table 1) learned a 30° counter-rotation (CR_wrist_) 5 min after R_wrist_ (Figure 1B). Performance of CR_wrist_ was worse than R_wrist_ (see Application of the Theory to the Experiments, in Results), clear evidence for anterograde interference by aftereffects from R_wrist_ onto CR_wrist_. Learning of CR_wrist_ 5 min after R_wrist_ caused catastrophic interference: performance on day 2 was not better than naive (Figure 1B and 1C). However, it appeared that there was an asymmetry in the savings and interference effects: savings, by definition, showed a marked improvement in learning (Figure 1A) whereas interference returned participants to a near naive state but not significantly worse (Figure 1B).

**Table 1:**
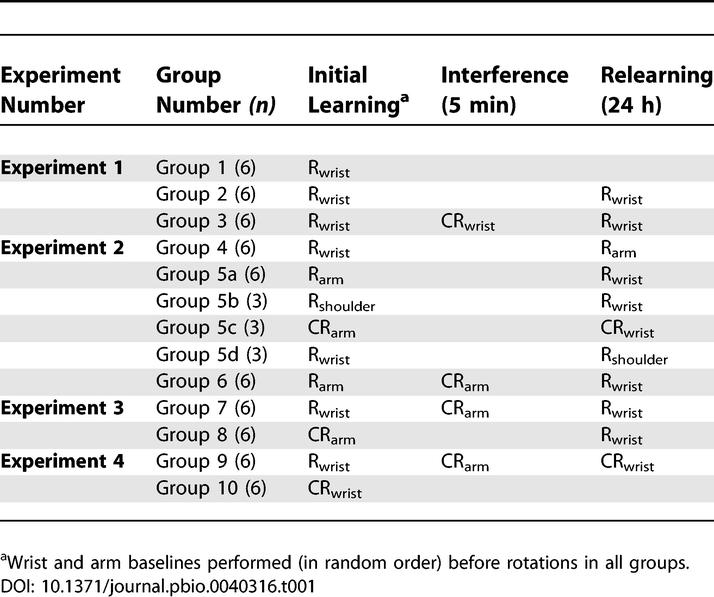
Participant Groups and Experimental Protocol

**Figure 1:**
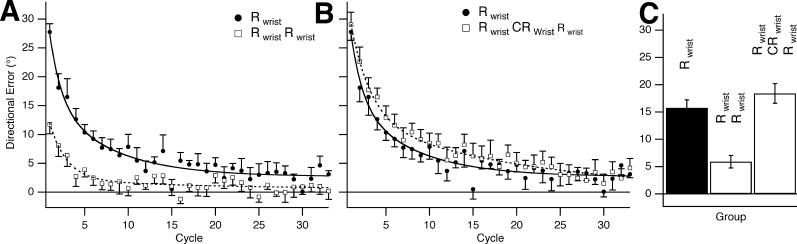
Savings and Interference Occur for Rotation Learning at the Wrist (A) R_wrist_ on day 1 (group 1, black circles and black curve) and on day 2 (group 2, white squares and dashed curve). Learning is shown by progressive reduction across cycles in the directional error at peak velocity. Points, representing the group average with standard error for each cycle, are fitted by a double-exponential function. There were substantial savings from day 1 to day 2. (B) R_wrist_ on day 1 (group 1, black circles and black curve) and after interference on day 2 (group 3, white squares and dashed curve). There were no savings from day 1 to day 2 after interference with CR_wrist_. (C) Bar graph showing a statistically significant difference in the reduction in mean directional error in the first six cycles for day 1 versus day 2 (groups 1 and 2, mean difference = 9.86°, *p* < 0.0001). This difference is absent with interference, with no statistically significant difference in the reduction in mean directional error in the first six cycles for day 1 versus day 2 (groups 1 and 3, mean difference = −2.64°, *p* = 0.22).

### Experiment 2a. Learning Was Slower with the Arm than with the Wrist

On day 1, some participants (group 4; Table 1) learned R_wrist_ while other participants (group 5a, Table 1) learned the same task with the arm (R_arm_). Interestingly, we found that learning with the arm was significantly slower than learning with the wrist (mean difference = 5.13°, *p* = 0.042). One possible explanation for this difference is that cursor feedback for the arm was veridical (it was projected on top of the hand) during unperturbed trials, whereas it was projected onto a vertical screen for the wrist. To control for this, we trained a separate group of participants (group 5b) to move the cursor on the vertical screen with shoulder movements alone, R_shoulder_ (no motion in elbow or wrist). Once again we observed that learning rates for the wrist were significantly faster than for the arm (mean difference = 6.46° per 6 cycles, *p* = 0.027).

### Experiment 2b. Learning Transferred from Arm to Wrist but Not from Wrist to Arm

On day 2, participants in each group re-learned with the other limb segment, i.e., those who had trained with the wrist were tested on the arm and vice versa (Figure 2A–2C). We found robust transfer from R_arm_ on day 1 to R_wrist_ on day 2 in group 5a (Figure 2A and 2C) and R_shoulder_ on day 1 to R_wrist_ on day 2 in group 5b (inset, Figure 2A). Similarly, there was transfer from counter-rotation at the arm (CR_arm_) on day 1 to CR_wrist_ on day 2 (group 5c), which was not significantly different from group 5a (mean difference = 0.475°, *p* = 0.857). Therefore, the degree of savings seen from arm to wrist was independent of the direction of the rotation and of whether we used a vertical screen or horizontal projection on top of the hand. In contrast, there was no significant transfer from R_wrist_ on day 1 to R_arm_ on day 2 (group 4; Figure 2B and 2C) or from R_wrist_ on day 1 to R_shoulder_ on day 2 (Group 5d; Figure 2B, inset). We ran a control study to check that the transfer from arm to wrist could be interfered with. As expected, transfer from R_arm_ to R_wrist_ was interfered with when CR_arm_ was learned 5 min after R_arm_ (group 6; Table 1): there was no significant difference between R_wrist_ on day 1 (group 1) and R_wrist_ on day 2 (group 6) (mean difference = −1.046, *p* = 0.6429). Thus, errors experienced with arm movements benefited subsequent learning with the wrist, but not vice versa. This result is congruent with previous reports of asymmetric transfer of prism adaptation [6,7].

**Figure 2:**
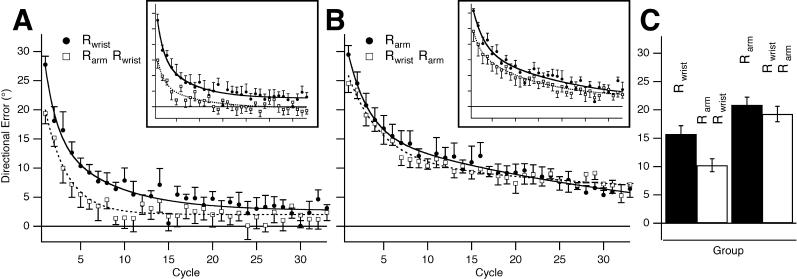
Savings Transfers from Arm to Wrist but Not from Wrist to Arm (A) R_wrist_ on day 1 (group 1, black circles and black curve) and R_wrist_ on day 2 after R_arm_ on day 1 (group 5a, white squares and dashed curve). There were substantial savings from R_arm_ on day 1 to R_wrist_ on day 2. Inset: R_wrist_ on day 1 (group 1, black circles and black curve) and R_wrist_ on day 2 after R_shoulder_ on day 1 (group 5b, white squares and dashed curve). There were substantial savings from R_shoulder_ on day 1 to R_wrist_ on day 2 (mean difference = 7.75° , *p* = 0.0157). Inset axes scaled as in main figure. (B) R_arm_ on day 1 (group 5, black circles and black curve) and R_arm_ on day 2 after R_wrist_ on day 1 (group 4, white squares and dashed curve). There were no significant savings from R_wrist_ on day 1 to R_arm_ on day 2. Inset: R_shoulder_ on day 1 (group 1, black circles and black curve) and R_wrist_ on day 2 after R_shoulder_ on day 1 (group 5b, white squares and dashed curve). There were no significant savings from R_wrist_ on day 1 to R_shoulder_ on day 2 (mean difference = 4.5° , *p* = 0.1). Inset axes scaled as in main figure. (C) First pair of bars showing a statistically significant difference in the reduction in mean directional error in the first six cycles for R_wrist_ on day 2 , after R_arm_ on day 1, compared to R_wrist_ on day 1 (groups 1 vs. 5a, mean difference = 5.52°, *p* = 0.01). Second pair of bars showing no statistically significant difference in the reduction in mean directional error in the first six cycles for R_arm_ on day 2, after R_wrist_ on day 1, compared to R_arm_ on day 1 (groups 5a versus group 4, mean difference = 3.52°, *p* = 0.12).

### Experiment 3. Prior Wrist Training Blocked Interference from Arm to Wrist

Experiment 1 established that savings and interference occurred for rotation learning with the wrist. In experiment 2, we found that learning at the arm transferred to the later testing with the wrist, which would suggest that learning of a counter-rotation at the arm would interfere with a prior memory acquired with the wrist. That is, if participants started with R_wrist_ training followed by CR_arm_ training, then there should be no savings on day 2 when participants were retested on R_wrist_. Contrary to this, and consistent with a contextual role for the effector used to learn the rotation, we found that there was significant savings for R_wrist_ on day 2 despite learning CR_arm_ only 5 min after R_wrist_ on day 1 (group 7; Table 1) (Figure 3A and 3B). Savings were not significantly different from those seen for R_wrist_ from day 1 to day 2 (*p* = 0.12). To exclude the possibility, albeit implausible, that savings for R_wrist_ resulted from learning of CR_arm_ rather than R_wrist_, we had a separate group of participants (group 8; Table 1) learn only CR_arm_ on day 1 and then R_wrist_ on day 2. As expected, no savings were found for R_wrist_ (Figure 3B). Thus, similar to the asymmetry of savings and interference effects seen in experiment 1, rotation learning at the arm transferred to the wrist, but counter-rotation learning at the arm did not make rotation learning at the wrist worse than naive. Similarly, there was no significant difference in performance between group 8 who learned CR_arm_ naive and group 7 who learned CR_arm_ 5 min after R_wrist_ (*p* = 0.8). Therefore, without the need for repeated alternation, participants learned and retained two opposite rotations within 5 min of each other nearly as well as if they had learned them separately.

**Figure 3:**
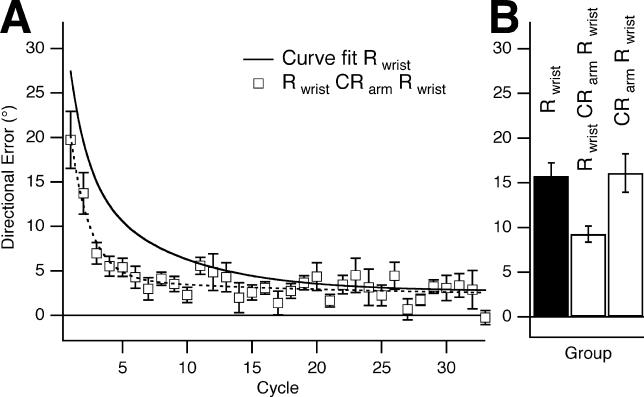
Rotation Learning at the Wrist Is Not Interfered With by Counter-Rotation Learning at the Arm (A) R_wrist_ on day 2, after R_wrist_ followed by CR_arm_ 5 min later on day 1 (group 7, white squares, dashed curve). There was savings from R_wrist_ on day 1 to R_wrist_ on day 2 despite CR_arm_. The thick black curve represents R_wrist_ on day 1 (group 1). (B) Bar graph showing a statistically significant difference in the reduction in mean directional error in the first six cycles for R_wrist_ on day 1 versus day 2 (groups 1 and 7, mean difference = 6.49°, *p* =0.0036). This difference was absent when only CR_arm_ was learned on day 1, with no statistically significant difference in the reduction in mean directional error in the first six cycles for day 1 versus day 2 (groups 1 and 8, mean difference = 0.328°, *p* = 0.88).

### Experiment 4. Prior Wrist Training Blocked Transfer from Arm to Wrist

In experiment 3, we showed that a change in effector could prevent interference: CR_arm_ did not interfere with prior learning of R_wrist_. In the final experiment, we asked whether, conversely, transfer from arm to wrist could be blocked by a prior history of wrist training. This experiment directly tests the hypothesis that interference seen in “A → B → A” experiments could be caused by an inhibitory context effect. This is because there is no a priori reason why the interference condition, if it is acting through a context effect, needs to be interposed between the other two. B → A → A should also lead to interference with re-learning of A.

Participants first learned R_wrist_ and then 5 min later learned CR_arm_ (group 9; Table 1). On day 2, they then learned CR_wrist_. We had shown in experiment 2 that R_arm_ transferred to R_wrist_ (group 5a), and, similarly, that CR_arm_ transferred to CR_wrist_ (group 5c). However, we found that transfer of savings from arm to wrist was blocked by previous experience of R_wrist_, i.e., learning R in the context of the wrist inhibited subsequent transfer of CR from the arm to the wrist (Figure 4A and 4B). In contrast to experiment 2, there was no transfer of CR_arm_ to CR_wrist_, and the learning rate of CR_wrist_ on day 2 was not significantly different than learning of CR_wrist_ on day 1 (group 10; Table 1). This failure to show transfer with CR_wrist_ is not due to learning of CR_wrist_ being somehow inherently more difficult than learning of R_wrist_, because there was no significant difference in the rate of learning R_wrist_ or CR_wrist_ on day 1 (comparing groups 1 and 10, *p* = 0.14), i.e., clockwise and counter-clockwise rotations are learned at the same rate. Thus, we found that learning of R_wrist_ did not interfere with subsequent learning of CR_arm_ and yet the prior learning of R_wrist_ prevented subsequent transfer from CR_arm_ to CR_wrist_. This result cannot be explained by either retrograde interference or by aftereffects. Instead, it strongly suggests that limb segment use acts as a contextual cue that blocks generalization.

**Figure 4:**
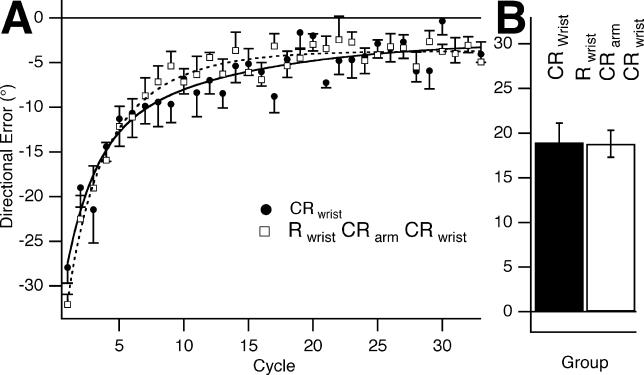
Antecedent Learning of the Rotation with the Wrist Blocks Subsequent Transfer of the Counter-Rotation from Arm to the Wrist (A) CR_wrist_ on day 2, after R_wrist_ followed by CR_arm_ 5 min later on day 1 (group 9, white squares, dashed curve). Also shown is CR_wrist_ on day 1 (group 10, black circles, black curve). There were no savings for CR_wrist_ on day 2 compared to CR_wrist_ on day 1. (B) Bar graph showing no statistically significant difference in the reduction in mean directional error in the first six cycles for CR_wrist_ on day 1 versus day 2 (groups 9 and 10, mean difference = −0.14°, *p* =0.947).

### A Statistical Model of Motor Adaptation with Contextual Cues

The experimental data produced three observations. First, visuomotor rotations associated with arm motion produced significantly slower adaptation rates than rotations associated with wrist motion. Second, training with the arm benefited subsequent learning with the wrist, but training with the wrist did not benefit learning with the arm. Finally, despite the fact that naive participants exhibited transfer from arm to wrist, this transfer was blocked if participants had prior training with the wrist (experiments 3 and 4). We will show that these results are generally consistent with a statistical formulation of the learning problem in which motor adaptation depends not just on the error in a given trial, but also on the prior history of training.

Participants were trained in two situations: with motion of the wrist, and with motion of the arm. In each case, a cursor indicated end-effector position (hand or finger). The computer imposed a perturbation of this position via a spatial rotation. Let us represent these positions in polar coordinates and focus only on their angular component. That is, if in trial *n* the end-effector angle is *e^(n)^* and the imposed rotation is *r^(n)^*, then the computer displays the cursor at *y^(n)^*

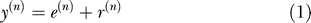
Now suppose that from the learner's point of view, the cursor angle that he observes is related to the angle of his end-effector, as well as a perturbation that depends on the context in which the end-effector was moved, plus some sensory noise. Let **c** *^(n)^* be a binary vector that specifies this context and **w** *^(n)^* be the weight vector that specifies the contribution of the context to the perturbation. That is, the learner hypothesizes that:

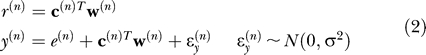
The term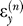 is a random variable that signifies noise in the sensory system of the learner and superscript *T* is the transpose operator. We assume that the sensory noise is normally distributed with mean zero and variance *σ* ^2^. Now suppose that the learner hypothesizes that perturbations are not permanent, and are affected by some noise themselves:

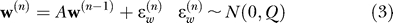
The term *A* is a constant and stable matrix, and expresses the belief that perturbations have a finite timescale. (A square matrix is considered to be stable if and only if the magnitudes of all the eigenvalues are smaller than one). The term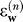 is a random vector that signifies noise that affects the perturbations. It is normally distributed with mean zero and diagonal variance-covariance matrix *Q*.

On trial *n*, the experimenter instructs the learner to move the end-effector to target location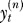 . To do so, the learner predicts the rotation that he expects will be present in this context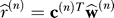 and moves the end-effector to cancel that perturbation:

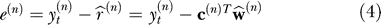
The experimenter provides feedback to the learner by displaying the cursor at position *y^(n)^*. The learner observes an error between the cursor position and the target,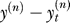 . For the learner, the objective is to minimize the expected value of the squared errors, i.e.,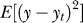 . This occurs when the learner minimizes the expected value of the squared difference between **w** and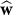 . The solution for this problem is an iterative algorithm described by Kalman [22].

On trial *n,* the learner has performed *n* − 1 trials and has observed the associated consequences *y^(n)^*. We use the term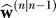 to label the learner's estimate on trial *n* based on the previous *n* − 1 observations. On trial *n,* based on this prior estimate, the learner moves the end-effector to location *e^(n)^*:

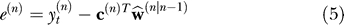
After the trial is complete, the learner observes *y^(n)^*. The difference between this position and the target is an error that the participant will learn from, resulting in a posterior estimate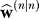 :

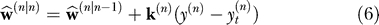
The vector **k** *^(n)^* is called the Kalman gain. It specifies how the error will affect the context in which it was experienced, and how the error will generalize to other contexts. The crucial idea is that this generalization is not arbitrary, but depends on the learner's uncertainty regarding his or her current parameter estimates. We label this uncertainty with matrix *P* and define it as the variance covariance of our parameter errors:

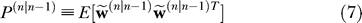
where the vector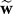 is defined as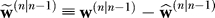 . The posterior estimate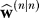 that minimizes the trace of matrix *P* is given by Equation 6 when the gain is set to:

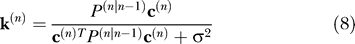
After observing *y^(n)^*, the posterior estimate will have the variance-covariance matrix described by:

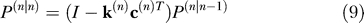



The learning rule in Equation 6 is equivalent to a Bayesian integration step. In this step, the learner weights her prior estimate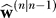 with uncertainty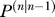 with the evidence observed in the current trial (Equation 2). The gain vector **k** expresses the optimal weighting of the two sources of information. We can simplify Equations 8 and 9 to produce a more intuitive formulation of the learning process:

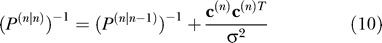


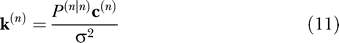
From Equation 11 we see that the learning gain depends on parameter uncertainty and this uncertainty depends on the history of contexts **c** *^(n)^* in which prior trials were performed (Equation 10). Therefore, the history of prior contexts crucially defines parameter uncertainty, which in turn defines the generalization pattern. Furthermore, increased uncertainty will result in increased sensitivity to error, and therefore faster learning. Our final step is to express the prior estimate in trial *n* + 1. Based on the hypothesis that we made about the learner in Equation 3, we have:

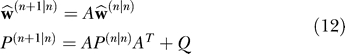



As an example, consider a scenario in which there are two contexts in which movements can be made (that is, **c** is a 2 × 1 binary vector). If both contexts are repeatedly present in a sequence of trials, then **c** *^(n)^* = [1 1]*^T^*, then the off-diagonal terms in the matrix *P* will become negative (Equation 10). Now, if in a given trial, only one cue is present, that is **c** *^(n)^* = [1 0]*^T^*, the Kalman gain will be a vector with a first term that is positive but a second term that is negative. As a result, the error in that trial will affect the estimate for both the context that is present and the context that is absent. In contrast, if the two contexts generally occur independently, then the off-diagonal terms in the uncertainty matrix will be close to zero. In this case, error experienced in one context will not generalize to the other context. We see that because parameter uncertainty depends on contextual history, sensitivity to error and its generalization will also be history dependent.

In summary, prior history plays a crucial role in the Bayesian learning process. In contrast, in LMS the parameter estimates for a context can change only when that context is present:

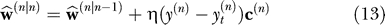
We will exploit this difference between LMS and Bayesian learning and show that the experimental data are generally consistent with a Bayesian learning process.

### Application of the Theory to the Experiments

The learner experienced errors in two situations: while moving the cursor with the shoulder and elbow joints of the arm, and while moving it only with the wrist. The arm motion did not involve motion of the wrist as viewed in proprioceptive coordinates. However, arm motion produced motion of the hand as viewed in an extrinsic space. In contrast, motion of the wrist did not involve motion of the upper arm in either extrinsic or intrinsic space. To explain the data, we need to make two crucial assumptions: First, let us assume that for the learner, the context is specified by whether a body part experienced motion in extrinsic space. That is, **c** *^(n)^* = [0 1]*^T^* if the trial involved only motion of the hand (i.e., a wrist trial), and **c** *^(n)^* = [1 1]*^T^* if the trial involved motion of both the hand and the upper arm (i.e., an arm trial). Second, we assumed that in daily activities of a typical participant, she is likely to experience coupled motion of the hand and upper arm. That is, when the upper arm moves, so does the hand (where motion is defined in extrinsic space).

To begin each simulation, we needed to specify the learner's prior. To produce the prior uncertainty matrix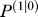 , we started from an arbitrary initial value and then assumed that before the participant had come to the lab and participated in the experiment, in 95% of “trials,” the learner had been in a context in which motion of the upper arm was accompanied with motion of the hand. That is, we used Equation 10 with the assumption that in 95% of trials, **c** *^(n)^* = [1 1]*^T^*, and in the remaining 5% of the trials, **c** *^(n)^* = [1 0]*^T^*. The prior uncertainty matrix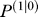 always converged to a matrix with negative off-diagonal elements (the actual value of the matrix depends on the measurement noise *σ* ^2^, which we arrived at by fitting to the measured data, see Materials and Methods). Furthermore, we assumed that at start of the experiment, the participant was naive about the rotations, i.e.,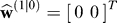 .

Let us consider R_wrist_ training. The experimenter sets *r* ^(*n*)^ = 30 and asks the learner to move the cursor with the wrist. The learner assumes that the context is **c** *^(n)^* = [0 1]*^T^*. Figure 5A shows the two components of the vector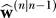 , i.e., the weight associated with the upper arm and the weight associated with the wrist. With each trial, the learner's estimate of the perturbation imposed on the wrist increases toward 30°. However, despite the fact that the context is wrist only, the estimate for the upper arm becomes negative, resulting in an estimate for the whole arm (upper arm + wrist) that is only slightly positive. Therefore, the model reproduces the result that wrist training will not have a significant impact on subsequent training with the arm. Prior training, in which most actions involved both motion of the upper arm and the wrist and therefore produced an uncertainty matrix with negative off-diagonal elements, is directly responsible for this generalization pattern.

**Figure 5:**
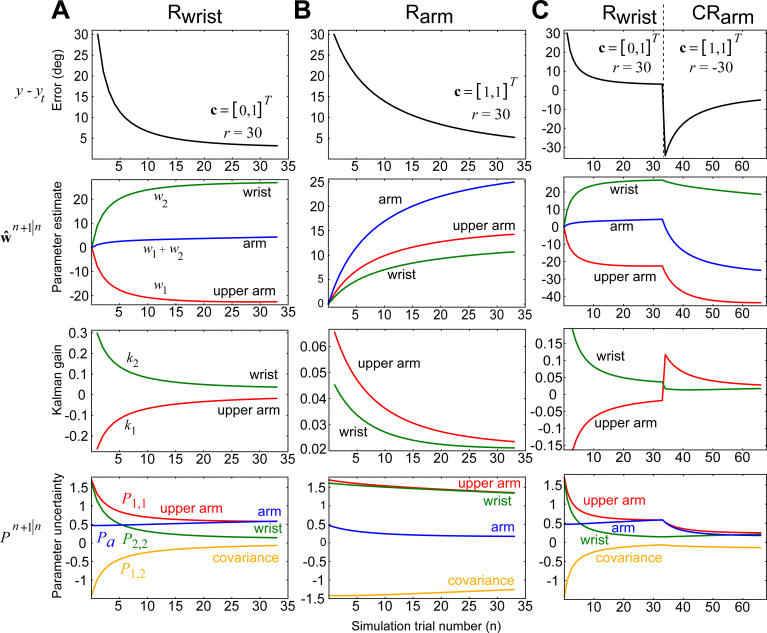
Simulation Results Each column displays the movement errors *y* ^(*n*)^ − *y_t_,* the two components of the parameter vector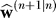 (and their linear combination), the two components of the Kalman gain vector **k** ^(*n*)^, and the components of parameter uncertainty matrix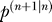 . For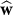 , the plot includes the upper arm estimate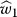 , the wrist estimate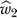 , and the arm estimate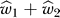 . For *P,* the plot includes the upper arm variance *P*_1,1_, the wrist variance *P*_2,2_, the covariance *P*_1,2_ (which is equal to *P*_2,1_), and the variance for the arm which is *P_a_* = *P*_1,1_ + *P*_2,2_ + *P*_1,2_ + *P*_2,1_. The context for each training situation is specified by the vector **c**. All simulations begin at the same initial conditions. (A) Simulation of R_wrist_. With each trial, the estimate for the wrist increases toward 30°. Despite the fact that only the wrist context is present, the estimate for the upper arm becomes negative. This is because the uncertainty matrix has negative off-diagonal elements *P*_1,2_, which arise from the prior assumption that motion of the upper arm usually results in motion of the wrist (in extrinsic space). (B) Simulation of R_arm_. Errors produce changes in the estimates of both the upper arm and the wrist, resulting in transfer to the wrist. Despite identical initial conditions, learning with the arm is slower than learning with the wrist. (In the subplots, the red line associated with the upper arm is hidden behind the green line associated with the wrist). (C) Simulation of R_wrist_ followed by CR_arm_. Despite the fact that in the naive condition, arm training transferred to the wrist (part B), prior wrist training blocked this transfer. By the end of training, the model acquired R at the wrist and CR at the arm. To see the reason for this, compare the Kalman gain at the start of arm training in this subplot with the same arm training in subplot B. In part C, gain for the upper arm is nearly twice as high as in part B. In contrast, in part C, the gain for the wrist is about half as high as in part B. The prior training with the wrist changed the pattern of generalization.

Next, consider R_arm_ training. Figure 5B shows the simulation results when we set *r* ^(*n*)^ = 30 and adapt in the arm context. When we set **c** *^(n)^* = [1 1]*^T^*, the observed errors will produce changes in estimates associated with both upper arm and the wrist, but because the covariance in the uncertainty matrix is negative, the learning gains (Kalman gain) are much smaller in the arm context than when the task is performed in the wrist context. Consequently, the arm context is learned more slowly. Despite the fact that the uncertainty matrix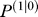 and the initial estimate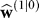 were identical in the two simulations of Figure 5A and 5B, the errors declined about twice as slowly in the context of the arm as compared to the wrist. Furthermore, the same uncertainty matrix dictates a generalization from arm to wrist, as the Kalman gain is positive for both the upper arm and wrist. As a consequence, arm training results in the estimate for the wrist to increases to about 15°. If we now test for R_wrist_, the wrist context has already learned half of the perturbation and will show transfer.

Let us now consider the observations made in experiments 3 and 4. We simulated initial training with the wrist on +30°, and then training with the arm in −30° (Figure 5C). The +30° wrist condition produced a −23° estimate for the upper arm. Now, when we simulated the arm −30° trials, the model showed a large change in the estimate for the upper arm but a small change in the estimate for the wrist. If we compare the Kalman gains for Figure 5C with Figure 5B, we see that if the wrist context precedes the arm context, then the generalization pattern of the arm context is significantly different. The extended wrist training increases the arm's uncertainty, making the gain for the upper arm about twice as large as for a naive participant. Therefore, when the arm context follows the wrist context, most of the error is now attributed to the upper arm (where the uncertainty is greatest). By end of the training, the arm is at −30°, but the wrist is still near +20°. Effectively, the model learns that each context produces a different estimate. Finally, for completeness, we also ran a simulation (unpublished data) to check the degree to which CR_wrist_ interferes with transfer of R_arm_ on day 1 to R_arm_ on day 2, and found savings close to that seen without intervening learning of CR_wrist_. This result is not unexpected given that, experimentally, wrist learning did not transfer to the arm.

To illustrate the model's strengths and weaknesses, in Figure 6 we plotted the data from all the experiments as well as the model's performance on each experiment. For example, in Figure 6A and 6B we re-plotted the data points in Figure 1A and 1B, but now the lines are model output rather than fits to the data. One of the strengths of the model is that it correctly produces learning with multiple timescales: the participants and the model are both very sensitive to error in initial trials of training, but then become less sensitive as trial numbers increase. This is because uncertainty tends to decrease with training (Figure 5A), which in turn makes the model less sensitive to prediction errors. Other strengths of the model include asymmetric transfer (Figure 6C and 6D), slower learning with the arm than with the wrist (Figure 6D and 6A), and history-dependent generalization from arm to wrist (Figure 6E).

**Figure 6:**
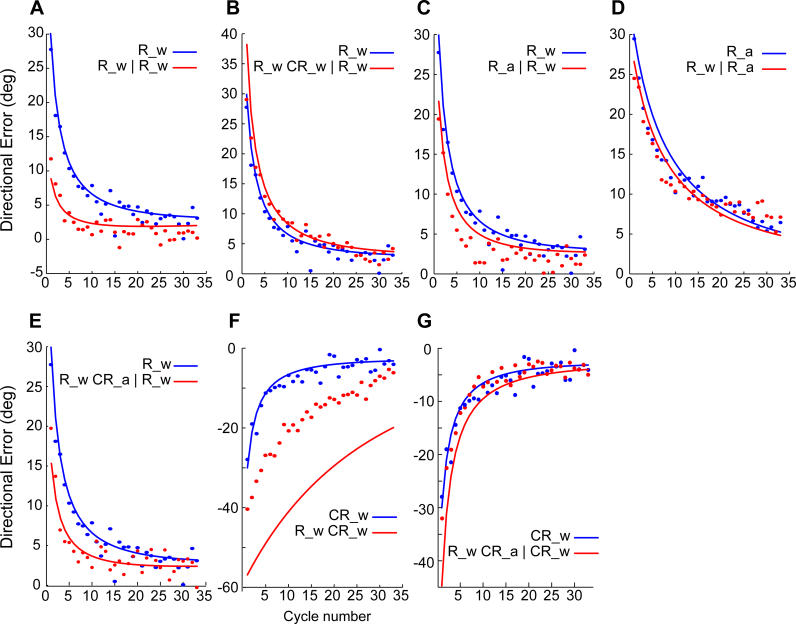
Simulation Results (Lines) along with the Measured Data (Dots) from All Experiments In the legend for each subplot, the vertical line refers to a 24-h break. (A) Savings from R_wrist_ day 1 to R_wrist_ day 2 (experiment 1). (B) Catastrophic interference when R_wrist_ on day 1 is followed by CR_wrist_ on day 1 and R_wrist_ is relearned on day 2 (experiment 1). (C) R_arm_ transfers to R_wrist_ (experiment 2). (D) Learning R_arm_ is slower than R_wrist_, and R_wrist_ shows little transfer to R_arm_ (experiment 2). (E) Prior learning of R_wrist_ blocks interference by CR_arm_ (experiment 3). (F) Learning of R_wrist_ interferes anterograde with CR_wrist_ learned 5 min later (experiment 3). (G) Prior learing of R_wrist_ blocks transfer of CR_arm_ to CR_wrist_ (experiment 4).

However, the model has important weaknesses. First, in experiment 1 when R_wrist_ was followed by CR_wrist_, the model predicted much stronger interference than we observed in our data (Figure 6F). The comparison of the data (red dots in Figure 6F) with the model (red line in Figure 6F) is interesting because the model correctly predicts that the rate of adaptation in CR_wrist_ (blue dots) will be much slower than in R_wrist_ (red dots). This is because training in R_wrist_ significantly reduces parameter uncertainty, resulting in slower learning in the subsequent CR_wrist_ training. Yet, the model cannot explain why initial performance (cycle 1) is so much better than expected. A likely possibility is that the 5 min of rest between the tasks produced some forgetting, something that we did not include in our model. Second, in experiment 4 when R_wrist_ was followed by CR_arm_, the model predicted moderately strong interference on subsequent testing on CR_wrist_ (the model predicted that performance on the first cycle should be significantly worse than observed). In contrast, the data (Figure 6G) showed no statistically significant evidence of worse performance for CR_wrist_, although transfer from CR_arm_ to CR_wrist_ was completely blocked. Again, the rate of adaptation was comparable in the model and the actual data. In these instances, the model predicted that prior training should have biased the learner, particularly in the first cycle. Yet the bias that we observed was generally smaller than expected.

## Discussion

When participants learned to control the trajectory of a rotated cursor with their arm or with their wrist, they exhibited complex patterns of behavior: They learned the arm task more slowly than the wrist. Their arm training generalized to the wrist, but the wrist training did not generalize to the arm. Finally, in participants that had prior training with the wrist, the expected generalization from the arm was blocked. Although the first two findings may seem like idiosyncrasies of generalization between limb segments, the third observation showed that a delta rule mechanism, which guides learning through gradual adjustments based only on recent errors, is inadequate to explain blocking of generalization across limb segments based on prior history of training. Instead, a “nonlinear” or context-based gating mechanism is suggested, in which history of limb segment use acts as the contextual cue. This history-dependent change in generalization allowed the participants to learn two distinct “maps” simultaneously: they learned a clockwise rotation with their wrist and a counter-clockwise rotation with their arm. In effect, they were able to “protect” their prior learning from subsequent generalization.

Why did the pattern of generalization change? Our thought was that generalization may depend on statistical properties of the task, which itself depends on the history of training. We imagined that the learner collected statistics on how the limb was used in the task, and generalized in order to minimize the expected value of his or her squared errors. A Bayesian description of the learning problem successfully predicted blocking of generalization based on prior limb segment use. Moreover, this model also predicted the previously unexplained proximal–distal asymmetry in transfer of learning. Thus, motor learning appeared to depend not only on motor error, but also on the history of prior actions.

### Transfer Was Asymmetric between Contexts

To manipulate context, we built upon our previous observation that visuomotor rotations are learned independently of novel dynamics [12]. Our data suggested that this was because rotations are learned by reducing visual errors whereas novel dynamics are learned proprioceptively. This led us to hypothesize that we could separate the visual error signal used to learn a new rotated mapping from the proprioceptive signal used to label a given context. The critical idea about context is that the contextual signal should be irrelevant to adaptation itself [23], which is the reason why arbitrary explicit cues, e.g., colors, have been so widely used experimentally. We chose a change in effector as the arbitrary contextual cue because we hypothesized that the adaptation-independent cue should be implicit rather than explicit. An important clue that this might indeed be the case comes from two observations. First, generalization of prism adaptation is velocity dependent [24], which suggests that the mapping is gated by the dynamic conditions under which it was learned. Second, a change in configuration of the arm allowed participants to eventually learn two opposing force fields [25]. However, interpretation of this second result is complicated by the fact that a change in configuration not only changes the context but also changes the force-field adaptation task itself, i.e., the same sensory signals provide error and context information. Thus, it cannot be concluded that the configuration change is purely a contextual effect.

In experiment 1 we showed that for naive participants, learning in the context of the wrist was faster than in the context of the arm. Furthermore, learning transferred from the arm to the wrist but not vice versa. Similar asymmetric transfer has been previously observed in prism adaptation. In that case, there was transfer from the shoulder to the wrist, but not from the wrist to the shoulder [6,7]. If we begin with the assumption that motion of the upper arm will inevitably result in motion of the wrist (or hand) in extrinsic space, then the model predicts both the observation that wrist learning will be faster than arm learning, and the asymmetric transfer from arm to wrist. It is of interest to ask how the contextual signal is conveyed. When participants learned the rotation with the arm, the wrist and fingers were immobilized with a splint, which means that there was no significant rotation of wrist joints to provide an intrinsic proprioceptive signal that correlated with cursor motion. Cursor motion was centered on the hand and the hand moved obligatorily with the arm. Thus during the arm context, both the upper arm and wrist moved in extrinsic coordinates. In contrast, when the rotation was learned around the wrist, the upper arm did not move in intrinsic or extrinsic coordinates. This leads to the novel idea that the relevant contextual cue is an implicit memory of motion of the limb segment in association with the reference frame in which prediction errors occurred. This still leaves unanswered what form the memory of limb motion takes. The memory is likely to have a proprioceptive component that identifies the motion as that of the whole arm or just the wrist. Interestingly, this memory might be fairly abstract because savings and interference for rotation learning can transfer across arms [26].

### A Change in Context Allowed Two Opposing Visuomotor Maps to Be Learned in Close Temporal Proximity

Experiment 3 was designed to test the prediction that identification of the right contextual cue would prevent generalization as interference between opposite visuomotor maps. We began with R_wrist_ training and then immediately trained participants in CR_arm_. Because in experiment 2 we had found that arm training transferred to the wrist, one might expect that CR_arm_ would catastrophically interfere with previous R_wrist_ training. However, we found that relearning of R_wrist_ showed a degree of savings comparable to when there was no intervening learning of CR_arm_. This result would not be expected if savings and interference were simply reciprocal processes based only on the direction of visual errors. Indeed, if this were the case, then an interference effect should have occurred when the rotation at the arm changed sign. Instead, savings were seen—the switch in effector led to dissociation between interference and savings effects. This result contrasts with previous attempts in recent years, largely unsuccessful, to identify contextual cues that will allow switching between visuomotor maps without interference. In a recent study using a joystick task, participants learned opposite 30° rotations within 15 min of each other [27]. Use of either a verbal or a color cue to separately identify the rotation and counter-rotation failed to prevent interference. A similar failure of color cues has been seen for larger rotations [28]. Similarly, attempts to prevent interference between opposing force fields with explicit symbolic cues have met with mixed success at best. In experiments in which participants alternated regularly between learning blocks of each force field, interference was not prevented by an explicit cue [25]. Monkeys were able to use a color cue to switch between viscous force fields but only after tens of thousands of trials of blocked training over several months [29]. Despite 3 d of training, human participants were unable to learn two randomly alternated force fields using color cues [30]. Another study found that this switching was possible only after very extensive training [31]. Better results were obtained when a change in arm configuration served as a cue to switch between two viscous force fields [25]. Our results in experiment 3 are quite distinct from these previous reports because interference was prevented at the first switch between rotation directions after an interval of only 5 min.

Experiment 4 was designed to complement experiment 3. It demonstrated that a contextual cue can also prevent generalization as transfer. Specifically, previous rotation training at the wrist prevented subsequent transfer of counter-rotation training from the arm to the wrist, transfer that would otherwise have occurred with savings at the wrist (experiment 1). The mechanism is not retrograde because R_wrist_ was learned before CR_wrist_. Nor is the mechanism an anterograde effect of R_wrist_ on CR_arm_, because in experiment 2, we had found that there was no significant transfer from wrist to arm. Finally, the result cannot be attributed to an anterograde effect of R_wrist_ on CR_wrist_, because we saw in experiment 1 that this does not lead to interference. This result provides an important clue as to why our previous study, and others like it, using the A_1st_ → B →A_2nd_ paradigm showed that CR_arm_ interferes with R_arm_ to the same degree when R_arm_ and CR_arm_ are separated by 24 h as when they are separated by only 5 min. Namely, if rotation direction changes but the context does not, i.e., always learned with the arm, then it is the last rotation learned in that context that is recalled. Therefore, consolidation, understood as stabilization of memory, may not be the process interfered with in many experiments that have used the A_1st_ → B →A_2nd_ paradigm, although consolidation of separate internal models almost certainly occurs, as we have demonstrated previously [11,32,33]. Instead, as mentioned in the introduction, we suggest that the failure to generalize seen with the A_1st_ → B →A_2nd_ paradigm [8–11] and in experiment 4 is caused by a powerful effect of context on retrieval of the correct rotation at re-learning. Critically, a contextual mechanism would show the order invariance we observed: B blocks transfer from A_1st_ to A_2nd_ as effectively with B→ A_1st_ →A_2nd_ (current results) as with A_1st_ → B →A_2nd_ (previous results). In both cases, use of the same limb segment context for rotation A and counter-rotation B is the key factor. Thus, our demonstration that history of training can alter patterns of generalization provides an important clue as to how the brain can recall different motor memories in rapid succession without interference.

The observations that history changed the patterns of generalization in experiments 3 and 4 were largely in agreement with the statistical model. Specifically, with prior training with the wrist, wrist estimates were hardly affected by subsequent learning of the counter-rotation with the arm. The reason for this was that training with the wrist affected the uncertainty associated with the upper arm. This in turn channeled most of the error to this part of the effector when the whole arm was subsequently used in the counter-rotation. As a result, after wrist and arm training, the model had acquired different maps for each context, despite the fact that in naive conditions, one context generalized to another. A fundamental property of the model was that parameter uncertainty depended on the history of contexts observed during training, not the history of errors. That is, the observed directional error by itself was not an effective contextual cue. Although in some cases of visuomotor adaptation, error itself can serve as a contextual cue [34], in our experiments, targets were presented randomly, which perhaps made consistent differentiation of clockwise and counter-clockwise errors difficult. Instead, what matters for our model is the history of limb contexts and correlations between them. The model suggests that patterns of generalization are a reflection of co-variance between the cues, consistent with the idea that the brain estimates second order statistics of action during motor learning.

In this task, it is of great interest that interference manifests as a return to naive and not worse than naive levels of performance. This is something that our model could not explain. It suggests that limb segment context causes retrieval of the congruent wrist rotation in experiment 3, but not of the incongruent wrist counter-rotation in experiment 4. Why this asymmetry? It can be speculated that there is transient retrieval of the counter-rotation, but this is rapidly suppressed when it does not lead to any reduction in prediction error. It brings to mind the architecture of learning suggested for a “mixture of experts” model, in which errors in prediction are used by a “moderator” to judge whether a contextually cued “expert” should be allowed to contribute to an output [35]. Once the expert is suppressed, both parameter estimates and uncertainty are reset to baseline levels, i.e., to the naive state.

### Conclusions

Our results demonstrate that an implicit memory of the limb segment used to learn a visuomotor mapping can serve as a contextual cue for recall of that mapping. The pattern of generalization across different contexts, either as transfer or interference, is not invariant, but rather is dependent on the history of training. When we consider the influence of prior training within the framework of statistical learning theory, what emerges is a motor system that learns not just from prediction error, but also from the history of implicitly remembered contexts in which training occurred.

## Materials and Methods

### Participants.

A total of 69 right-handed participants (33 men and 36 women, mean age of 29.2 ±6.4 y) participated in the study. All participants were naive to the purpose of the experiments, signed an institutionally approved consent form, and were paid to participate. There were four experiments, and different participants were randomly assigned to a particular group within each experiment (13 groups in total) (Table 1).

### Experimental protocol—arm apparatus.

Participants sat and moved a hand cursor by making planar reaching movements of the shoulder and elbow over a horizontal surface; positioned at shoulder level. The targets and the start point were projected onto a computer screen positioned above the arm. A mirror, positioned halfway between the computer screen and the table surface, reflected the computer display, producing a virtual image of the screen cursor and targets in the horizontal plane of the fingertip. Hand positions, calibrated to the position of the fingertip, were monitored using a Flock of Birds (Ascension Technology, Burlington, Vermont, United States) magnetic movement recording system at a frequency of 120 Hz. Anterior–posterior translation of the shoulder was prevented with a rigid frame around the trunk. The wrist, hand, and fingers were immobilized with a splint and the forearm supported on an air-sled system. An opaque shield prevented participants seeing their arms and hands at all times.

### Experimental protocol—wrist apparatus.

Participants sat in a chair and made pointing movements through combinations of abduction–adduction and flexion–extension movements around the wrist, so as to point their index finger at targets projected onto a vertical computer screen. Supination and pronation of the wrist was prevented with a rigid splint. The participant.s right hand was lightly taped in a fist position using medical paper tape, and a 1.5-cm spherical reflective marker was attached to the tape and positioned over the index finger's first interphalangeal joint. The hand was hidden from view. The position of the marker was monitored using a Qualysis ProReflex video camera (model MCU 240; Qualisys, Gothenburg, Sweden) equipped with an infrared strobe coupled to a video digitizer, which records the marker's position in the vertical plane with a spatial resolution of less than 1 mm at a frequency of 100 Hz. Hand position was ported to a Macintosh PowerMac G4 computer (Apple, Cupertino, California, United States) running custom software, which acquired data, controlled experiments, and updated the display in real time so that participants had continuous feedback of wrist position visible as a black cursor on the vertical computer screen.

### Experimental protocol—general protocol.

Experimental sessions were run over two consecutive days (day 1 and day 2). Targets were presented in blocks of 11 cycles of eight targets. Participants were instructed to make straight out-and-back movements with a sharp reversal within the target. To ensure that movements were made fast and to minimize on-line corrections, the black cursor disappeared after 150 ms and the reversal point was indicated by a white square [36].

On day 1, participants were first familiarized with baseline blocks (no rotation imposed) with the wrist and/or arm apparatus, depending on which experimental group they were in. Subsequently, participants performed three training blocks of a rotation (R), in which the screen cursor was rotated 30° counter-clockwise around the center of the start location. After a delay of 5 min, certain groups of participants performed three blocks of the counter-rotation (CR), in which the screen cursor was rotated 30° clockwise. On day 2, participants re-learned R.

### Experimental protocol—data analysis.

For each movement, peak velocity and reversal points were calculated as reported previously [3]. We used the directional error at the peak velocity as the measure of rotation adaptation. To assess the time course of adaptation to the imposed rotations, we computed the mean directional error over the first six cycles of eight movements. Differences between groups were assessed by comparing the six-cycle measure across groups by analysis of variance. Pair-wise post-hoc tests were performed with the Fisher PLSD (protected least significant differences) with a significance level of 0.05.

### Modeling.

There were four important parameters in the model, and we began by setting these parameters to very general values that were not informed by specific data. The first three parameters were the state transition matrix *A* in Equation 3, reflecting how much the learner forgets from trial to trial, and the state and measurement noises in Equations 2 and 3. We set these values as follows: *A* = 0.99*I, Q* = 0.5*I,* and *σ* ^2^ = 1, where *I* is a 2 × 2 identity matrix. The fourth parameter was *A* ^*^, which described how much participants forgot from end of training in day 1 to start of testing on day 2. We set *A* ^*^ = 0.80*I*. All simulations began with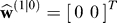 . Eq. (10) suggests that if we know the measurement noise, then we can estimate the prior by assuming a particular contextual history. The prior uncertainty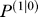 was acquired by starting at a random initial condition and iterating until convergence under the assumption that before the participant came to the lab, in 95% of “trials.” motion of the upper arm was coincident with motion of the hand, i.e., **c** *^(n)^* = [1 1]*^T^*. For the remaining 5% of trials we set **c** *^(n)^* = [0 1]*^T^*, i.e., wrist moved without motion of the upper arm. In no trial did the upper arm move without also moving the wrist.

This very general start was sufficient to reproduce all the patterns that are exhibited in Figure 5, i.e., learning curves that exhibit multiple timescales, faster wrist learning than arm learning, asymmetric transfer from arm to wrist, and blocking of transfer with prior training in the wrist. All these properties except the first one arise from the shape of the uncertainty matrix, which is directly due to our assumption that prior actions included mostly conditions where motion of the upper arm also moved the wrist. The multiple timescales arise from the Bayesian formulation of learning (Equations 10 and 12), in which uncertainty tends to decrease with increased observations.

To find the model parameters that were matched to the actual data, we fitted the model simultaneously to the measured performances in groups 1, 2, 3, 4, 5a, and 7. In our simulations, each “cycle” was one trial. We optimized the parameter values by minimizing the sum of squared errors between the model predictions and the experimental data (MATLAB optimization function lsqnonlin). We arrived at the following values: *A* = 0.9968*I*, A^*^ = 0.79*I*, *Q* = 0.0041*I*, *σ* ^2^ = 3.8, and *P* = [1.7 −1.4; −1.4 1.6]. These values were used for the simulations shown in Figures 5 and 6.

## Abbreviations

CR_arm_: counter-rotation at the arm
CR_wrist_: counter-rotation at the wrist
R_arm_: rotation at the arm
R_shoulder_: rotation at the shoulder alone
R_wrist_: rotation at the wrist
